# Metabolic heterogeneity affects trastuzumab response and survival in HER2-positive advanced gastric cancer

**DOI:** 10.1038/s41416-023-02559-6

**Published:** 2024-01-24

**Authors:** Jun Wang, Na Sun, Thomas Kunzke, Jian Shen, Annette Feuchtinger, Qian Wang, Raphael Meixner, Ronan Le Gleut, Ivonne Haffner, Birgit Luber, Florian Lordick, Axel Walch

**Affiliations:** 1 Research Unit Analytical Pathology, Helmholtz Zentrum München – German Research Center for Environmental Health, Neuherberg, Germany; 2 Core Facility Statistical Consulting, Helmholtz Zentrum München, 85764 Neuherberg, Germany; 3 University Cancer Center Leipzig (UCCL), Leipzig University Medical Center, Leipzig, Germany; 4 Technische Universität München, Fakultät für Medizin, Klinikum rechts der Isar, Institut für Allgemeine Pathologie und Pathologische Anatomie, München, Germany; 5 Department of Oncology, Gastroenterology, Hepatology, Pulmonology and Infectious Diseases, Leipzig University Medical Center, Leipzig, Germany

**Keywords:** Gastric cancer, Cancer metabolism, Tumour heterogeneity, Targeted therapies

## Abstract

**Background:**

Trastuzumab is the only first-line treatment targeted against the human epidermal growth factor receptor 2 (HER2) approved for patients with HER2-positive advanced gastric cancer. The impact of metabolic heterogeneity on trastuzumab treatment efficacy remains unclear.

**Methods:**

Spatial metabolomics via high mass resolution imaging mass spectrometry was performed in pretherapeutic biopsies of patients with HER2-positive advanced gastric cancer in a prospective multicentre observational study. The mass spectra, representing the metabolic heterogeneity within tumour areas, were grouped by *K*-means clustering algorithm. Simpson’s diversity index was applied to compare the metabolic heterogeneity level of individual patients.

**Results:**

Clustering analysis revealed metabolic heterogeneity in HER2-positive gastric cancer patients and uncovered nine tumour subpopulations. High metabolic heterogeneity was shown as a factor indicating sensitivity to trastuzumab (*p* = 0.008) and favourable prognosis at trend level. Two of the nine tumour subpopulations associated with favourable prognosis and trastuzumab sensitivity, and one subpopulation associated with poor prognosis and trastuzumab resistance.

**Conclusions:**

This work revealed that tumour metabolic heterogeneity associated with prognosis and trastuzumab response based on tissue metabolomics of HER2-positive gastric cancer. Tumour metabolic subpopulations may provide an association with trastuzumab therapy efficacy.

**Clinical trial registration:**

The patient cohort was conducted from a multicentre observational study (VARIANZ;NCT02305043).

## Background

Gastric cancer (GC) is currently the fourth most common cause of cancer-related deaths globally [1]. Trastuzumab, a recombinant humanised monoclonal antibody directed against the human epidermal growth factor receptor 2 (HER2), is the only targeted agent approved for the first-line treatment of patients with HER2-positive advanced GC [2]. Trastuzumab combined with platin–fluoropyrimidine chemotherapy improves survival outcomes in HER2-positive GC [2]. Nevertheless, only a subgroup benefits from the addition of trastuzumab to chemotherapy. The overall response rate of the combined therapy is less than 50%, indicating that a considerable proportion of HER2-positive cancers are resistant to HER2 inhibition [3]. Optimising the selection of HER2-targeted regimens by identifying patient subpopulations who would benefit from trastuzumab could be cost-effective and would spare some patients unnecessary exposure to ineffective treatments.

Molecular heterogeneity exhibits a variety of biological behaviours in cancers [4]. Exploring the patterns of molecular heterogeneity are necessary to design personalised targeted regimens to increase patient response [5–8]. GC has a high level of genomic and phenotypic variability even within individual tumours, and this underlying heterogeneity is considered as a major cause for the frequent failure of biomarker-based clinical trials [9–11]. High incidence of HER2 heterogeneity was observed in GC and it was associated with chemotherapy [12] and trastuzumab efficacy [13]. Several studies uncovered proteomic subpopulations that were linked to patient survival in GC [14–16]. Metabolic reprogramming has been recognised as one hallmark that can be used to prevent therapeutic resistance [17]. Metabolomics, a predictor of drug therapeutic response in cancers [18, 19], can generate metabolite profiles and also combine this information with changes in crucial metabolic pathways, such as Warburg effect, altered amino acid/lipid/drug metabolism, generation of drug-resistant cancer stem cells, and immunosuppressive metabolism [17]. Metabolite profile was considered an important factor besides HER2 status in assessing the initial response to trastuzumab treatment for GC patients [20, 21]. Specifically, one study revealed tumour metabolic heterogeneity within HER2/neu-positive and HER2/neu-negative GC cells [22]. Nonetheless, the impact of intratumoural and intertumoural metabolic heterogeneity on trastuzumab response in HER2-positive advanced GC remains unclear. Matrix-assisted laser desorption/ionisation–imaging mass spectrometry (MALDI–IMS) enables the imaging of different molecular classes in their histopathological context and thus the allocation of molecular profiles to specific tumour cell types [23–25]. This high cellular specificity is behind the increasing popularity of IMS and its proven ability to identify diagnostic and prognostic biomarkers [26–28]. Additionally, MALDI–IMS is an omics technique that allows for the global characterisation of the spatial metabolomics [29, 30], which offers an opportunity to demonstrate the drug-resistant tumour profile with metabolic heterogeneity and discovering the alteration in the tumour microenvironment [17]. Combined with statistical tools, MALDI-IMS constitutes a unique tool to reveal a priori tumour subpopulations that are not distinguishable using conventional histopathological methods, but which are molecularly distinct [31–33].

**Fig. 1:**
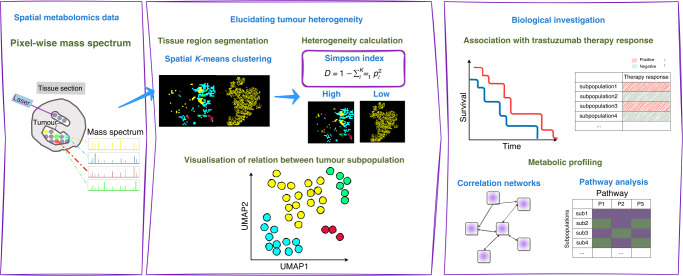
Schematic overview of the de novo identification of metabolic heterogeneity and tumour subpopulations. The workflow comprises approaches used to assess metabolic heterogeneity and tumour subpopulations in patients with HER2-positive gastric cancer, followed by bioinformative analyses linked to clinical data.

We apply spatial metabolomics and *K*-means clustering method to identify metabolically distinct tumour subpopulations of HER2-positive advanced GC from routinely preserved pretherapeutic biopsies, and assess their relationships with the response to trastuzumab treatment. The workflow of this study is shown in Fig. 1.

## Materials and methods

### Trastuzumab-treated advanced GC patient cohort

Patients receiving medical treatment for histologically confirmed metastatic GC (including oesophagogastric junction cancer, all Union for International Cancer Control (UICC) stage IV) were recruited after providing written informed consent. The patient cohort was conducted from a multicentre observational study (VARIANZ; NCT02305043) in accordance with the Declaration of Helsinki [34]. Approvals of the ethics committees of Leipzig University Medical Faculty and all participating centres were obtained before site activation. All patients included in this analysis were HER2-positive and underwent trastuzumab therapy and chemotherapy. The cohort was divided into therapy-resistant (*n* = 17) and therapy-sensitive (*n* = 25) patients by overall survival (cutoff = 13.8 months, Gastric Cancer (ToGA) trial [2]). Table 1 shows the clinical characteristics of all included patients.

**Table 1:** Summary of patient characteristics.

Characteristic	
Number of patients	49
Age [years]	
Median	66
Range	24–89
Gender	
Male	38
Female	11
UICC stage	
IV	49
Survival time [months]	
Median	14
Range	0–80
HER2 IHC score	
0+	0
1+	0
2+	9
3+	40
*HER2* CISH	
Amplified	47
Non-amplified	0
n.a.	2

Samples with insufficient data to make a conclusion were set to ‘n.a.’.

### Central HER2 testing

GC HER2 status data were obtained from the previous study [21]. In brief, HER2 status was determined according to published standards in the central pathology using immunohistochemistry (IHC) and chromogenic in situ hybridisation (CISH) [34].

### High mass resolution MALDI–Fourier transform ion cyclotron resonance (FT–ICR) IMS

Data for spatial metabolomics were obtained from the previous study [21]. Formalin-fixed, paraffin-embedded (FFPE) biopsies coated with 9-aminoacridine (9-AA) hydrochloride monohydrate matrix (Sigma-Aldrich) were analysed in the negative ion mode on a Bruker Solarix 7.0 T FT–ICR MS (Bruker Daltonik) over a mass range of *m*/*z* 50–1000 as previously described [35]. After the acquisition, samples were stained with haematoxylin and eosin (H&E), coverslipped, scanned with a Mirax Desk scanner (Zeiss, Göttingen, Germany), and coregistered with the respective IMS data using flexImaging™ v. 4.0 (Bruker). Tissues were processed using virtual microdissection with the definitions of the regions of interest as tumour cells.

### Unsupervised identification of heterogeneity and Simpson’s diversity index calculation

The software SCiLS Lab (2020b Pro) was used for unsupervised segmentation of the MALDI-IMS data. MALDI-IMS raw data were first imported into the SCiLS Lab software. The standard segmentation pipeline then starts with data preprocessing, including baseline removal, normalisation, and peak selection. Hereafter, edge-preserving image denoising was carried out. At this stage, we consider a MALDI-imaging data set as a datacube with 3-coordinates: *x*, *y*, and *m*/*z*. Given the *m*/*z* value, an image of intensities of all spectra at this *m*/*z* value can be reconstructed. The resulting denoised data were spatially segmented using the *k* means algorithm with *K* ranging from 2 to 10. For *k*-means clustering, we used the correlation distance. The created segmentation maps with nine subpopulations were then used to identify areas in which similar spectra occur across the patients. Simpson’s diversity index was calculated for each of the patients based on the number of pixels in each of the subpopulations. It measures the diversity and indicates the probability that two randomly chosen pixels are from different subpopulations. It is defined as follows $D=1-\mathop{\sum }\nolimits_{i=1}^{K}{p}_{i}^{2}$, where ${p}_{i}$ is the share of pixels in subpopulation $i$ and ${K}$ is the number of clusters [36]. It can have values between 0 and 1; The higher the values of the index indicates the higher the diversity of the pixels in the different subpopulations for a patient.

### Cluster presence threshold optimisation and Cox proportional hazards regression model

The statistical analysis required linking the survival data of samples to the presence of specific clusters (subpopulations). To do so, a sample was assigned to a cluster if the cluster was sufficiently present in that sample, i.e. if the cluster held a higher fraction of pixels than a certain threshold (termed cluster presence threshold). A single sample could be assigned to more than one cluster if it contained significant tumour heterogeneity. The effect of choosing different thresholds on survival was investigated using Cox proportional hazards regression model as previously described [14]. In brief, an iterative loop was created with thresholds. At each threshold, a binary variable was created by applying the threshold to the cluster ratio. Cox proportional hazards regression model was then built using the thresholded data. The quality of each regression model was evaluated using the Akaike information criterion (AIC) [14]. The AIC provided a measure of model goodness of fit, and the preferred model was the one with the lowest AIC value over all values of *K* [2–10] and threshold (4–40%).

### Pathway analysis and correlation network analysis

Metabolites were annotated with the Kyoto Encyclopaedia of Genes and Genomes (KEGG; www.genome.jp/kegg/), allowing M-H, M-H_2_O-H, M + K-2H, M+Na-2H, and M+Cl as negative adducts. The altered metabolites in each subpopulation were identified by comparing with the other subtypes using the Mann–Whitney *U*-test with a cutoff adjusted *p* value < 0.05 and a fold change of 1.5. Pathway analysis was performed via the KEGG database using the MetaboAnalyst online tool (www.metaboanalyst.ca; Fisher’s exact test, *q* < 0.05 for FDR correction). Correlation networks were created based on the above significant metabolites using Cytoscape (v. 3.8.0) [37]. All networks were visualised using the absolute value of the correlation coefficient calculated by Spearman’s rank-order correlation.

### Statistical analysis

Patients’ subpopulations survival was compared with Kaplan–Meier curve. Accounting for multiple measurements for one patient due to several subpopulations, a count process following the formulation of Andersen and Gill was used. The Wald test was used to determine statistical difference in survival. The *p*-values are non-adjusted due to the limited sample size. To investigate the association between survival time and heterogeneity level of patients, cutoff-optimised survival analyses were performed, which in this context means that the threshold for low and high heterogeneity (cutoff = 0.068) was chosen such that the *p* value by log-rank test is minimal (*p* = 0.002), while ensuring robust results for similar cutoffs. Correlations were calculated using a pairwise Spearman rank-order correlation with non-adjusted *p* values. The Fligner–Killeen test was used to compare the variances of metabolites, and the Mann–Whitney *U*-test was used to determine the significantly altered metabolites for each subpopulation and the calculated *p* values were adjusted using the Benjamini–Hochberg correction. Two sided *p* values < 0.05 were considered statistically significant.

## Results

### Tumour metabolic subpopulations identification and metabolic heterogeneity visualisation

**Fig. 2:**
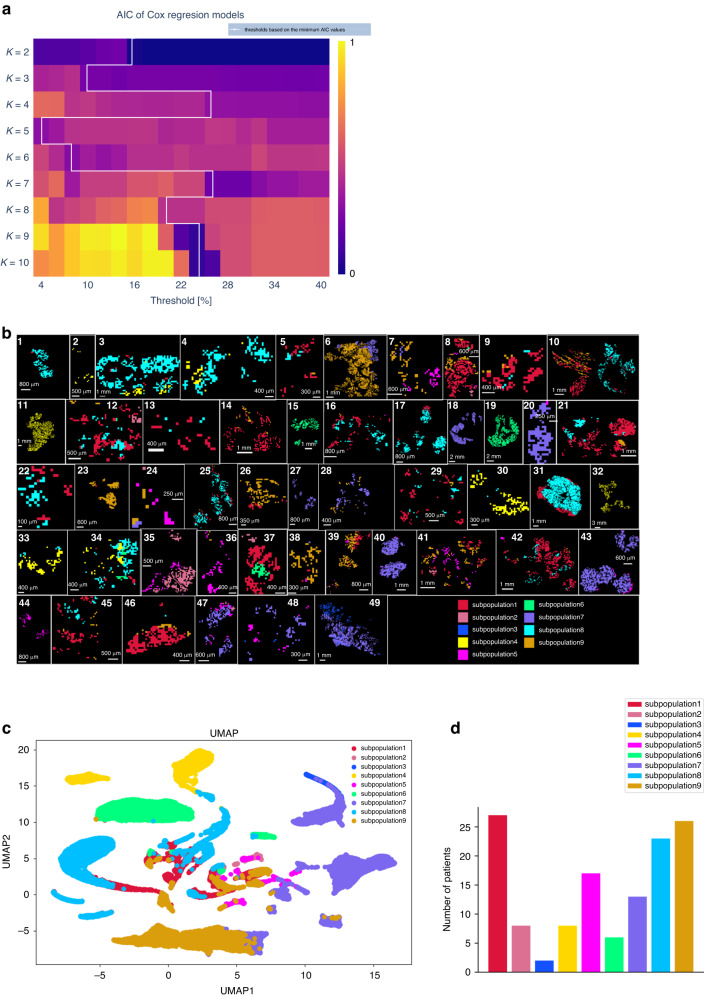
*K*-means unsupervised clustering analysis identifies nine tumour metabolic subpopulations. **a** Optimisation of the cluster presence threshold from *K* = 2 to 10 for comparison with clinical endpoint assessed by the Akaike Information Criterion (AIC) of Cox proportional hazards regression models. The AIC values were scaled to 0–1 and visualised as in a heatmap. Cluster numbers of *K* = 9 and *K* = 10 show the lowest AIC value at a threshold of 24%, and thus are defined as the optimal number of clusters. All the subsequent analysis was based on *K* = 9. **b** Mass spectra and ion distribution maps based on nine subpopulations of metabolites as generated by *K*-means analysis. *K*-means image was created by labelling each pixel according to its subpopulation label. **c** Uniform Manifold Approximation and Projection (UMAP) analysis of nine subpopulations. The points represent samples and are coloured by the tumour subpopulation label of each pixel. **d** Bar plot of the number of patients contributing to each of the subpopulations.

First, *K*-means clustering with *K* ranging from 2 to 10 was applied to identify survival-associated tumour subpopulations. Cox proportional hazards regression model for each of the K clusters was applied to identify the optimal number of clusters after assigning different subpopulation presence thresholds ranging from 4 to 40% [14]; 4–26% thresholds result in the best regression models. *K* = 9 and *K* = 10 clusters with a threshold of 24% have the lowest Akaike Information Criterion (AIC) value from the Cox regression models across all *K* values and thresholds, and are thus defined as the optimal values for the number of clusters and the threshold (Fig. 2a). Following the principle of parsimony, all the subsequent analysis was based on *K* = 9. To estimate the ability of metabolomics data to distinguish tumour subpopulations, the *K*-means image for the distribution of nine subpopulations is shown in the unsupervised segmentation image, revealing the tumour metabolic heterogeneity within patients (Fig. 2b). Figure 2c indicates that subpopulation 4, 6, 7 and 9 could be clearly separated by Uniform Manifold Approximation and Projection (UMAP) analyses based on the abundances of metabolites. Figure 2d shows the number of patients in each tumour subpopulation.

### Simpson’s diversity index reveals that patients with high metabolic heterogeneity have a better prognosis

**Fig. 3:**
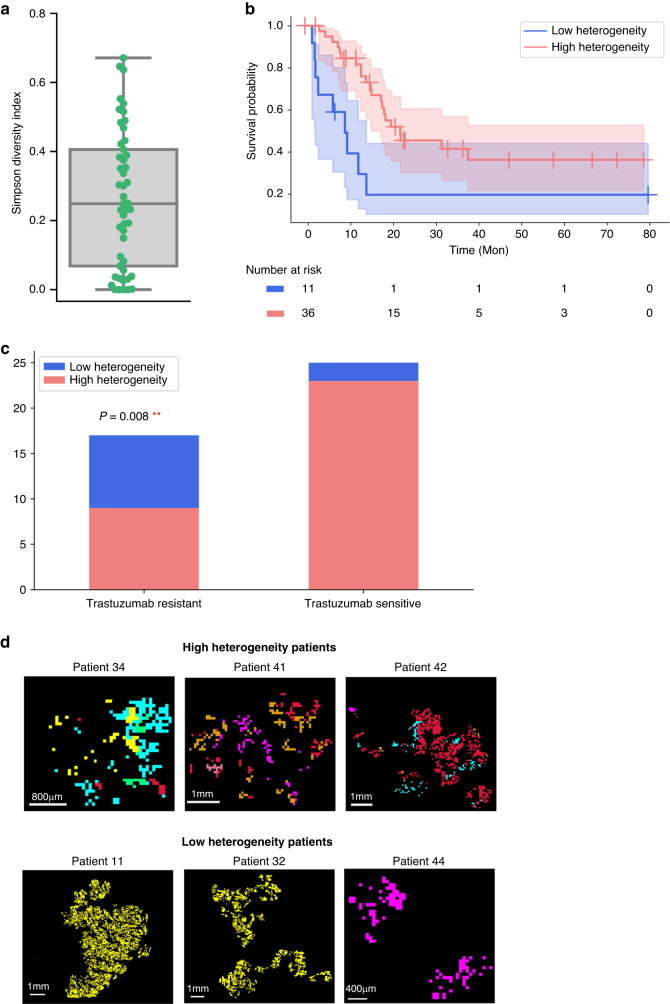
High heterogeneity patients have a better prognosis. **a** Simpson’s diversity index for *K* = 9. Higher Simpson’s diversity index value indicates higher heterogeneity level of the patient. The line within the box displays the median value. **b** Survival difference between patients with high metabolic heterogeneity levels and patients with low metabolic heterogeneity levels. **c** Numbers of high heterogeneity and low heterogeneity patients in trastuzumab-sensitive and trastuzumab-resistant patients. *p* value was calculated using the Fisher exact test. **d** Representative higher-magnification ion distribution images of three high- and low-heterogeneity patients within the measured tumour areas. **p < 0.01.

To further compare the heterogeneity level of individual patients, Simpson’s diversity index was applied. Figure 3a shows the Simpson’s diversity index calculated for each patient using the nine subpopulations from the *K*-means analysis. Patients with high Simpson’s diversity scores are associated with better patient outcomes (Fig. 3b). Additionally, the percentage of high heterogeneity patients is significantly higher in the trastuzumab-sensitive patients (82%) than in the trastuzumab-resistant patients (44%) (*p* = 0.008) (Fig. 3c). Overall, these analyses demonstrate the potential relation of tumour heterogeneity with survival and trastuzumab therapy in HER2-positive GC. Figure 3d shows the representative ion distribution maps of patients with high and low heterogeneity.

### Clinical impact of tumour metabolic subpopulations on survival and trastuzumab efficacy

**Fig. 4:**
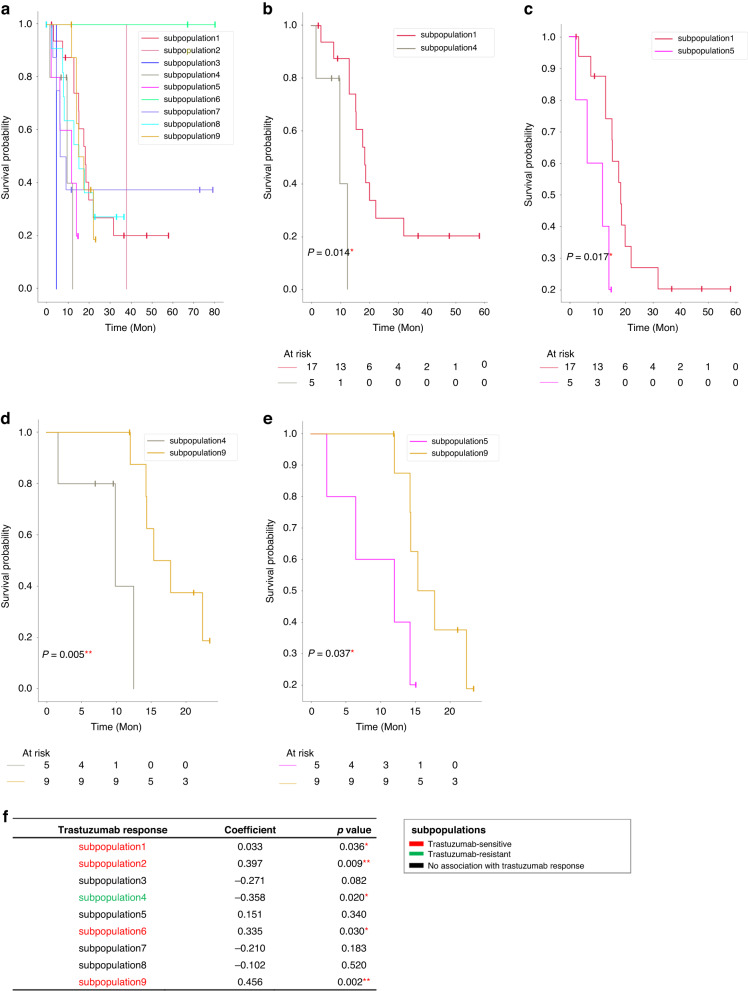
Tumour metabolic subpopulations are associated with patient survival outcomes and trastuzumab therapy response. Survival analysis of **a** nine tumour metabolic subpopulations in Kaplan–Meier curves. In pairwise comparison, there are significant differences in survival between **b** subpopulations 1 and 4, **c** subpopulations 1 and 5, **d** subpopulations 4 and 9, and **e** subpopulations 5 and 9 by Wald test. **f** Association of nine tumour metabolic subpopulations with trastuzumab therapy response. The subpopulations which positively correlated with trastuzumab response were defined as trastuzumab-sensitive subpopulations; The subpopulation which negatively correlated with trastuzumab response was defined as trastuzumab-resistant subpopulation. *$p$ < 0.05, **$p$ < 0.01.

After applying the AIC-optimised threshold for tumour subpopulations, Kaplan–Meier curves of the nine subpopulations are compared (Fig. 4a). In the pairwise comparisons, there is a significant difference in survival between the subpopulations encompassed by subpopulations 1 and 4 ($p$ = 0.014), and subpopulations 1 and 5 ($p$ = 0.017), respectively (Fig. 4b, c). The Kaplan–Meier curve indicates better outcomes for subpopulation 9 than both subpopulations 4 ($p$ = 0.005) and 5 ($p$ = 0.037) (Fig. 4d, e). In order to confirm the clinical importance of the tumour subpopulations, we identically performed the survival analysis for another two values of *k* (*K* = 10 and *K* = 5) for the GC MSI dataset, and compared them with *K* = 9. As shown in Supplementary Fig. 1, clinical consistency of these subpopulations indicates their robustness towards changing of *K*. There are no statistically significant differences in other pairwise tumour subpopulation comparisons. Spearman correlation analysis was performed to investigate the association of each of the nine tumour subpopulations with the trastuzumab response. The result shows that subpopulation 1 ($p$=0.036), subpopulation 2 ($p$=0.009), subpopulation 6 ($p$=0.030), while subpopulation 9 ($p$=0.002) are associated with trastuzumab sensitivity, and subpopulation 4 is associated with trastuzumab resistance ($p$=0.020). There is no correlation between trastuzumab efficacy and subpopulations 3, 5, 7, and 8 (Fig. 4f). Taken together, we find that tumour subpopulations 1 and 9 are associated with favourable prognosis and trastuzumab sensitivity, and subpopulation 4 is associated with poor prognosis and trastuzumab resistance.

### Potential metabolites and pathways associated with trastuzumab therapy response

**Fig. 5:**
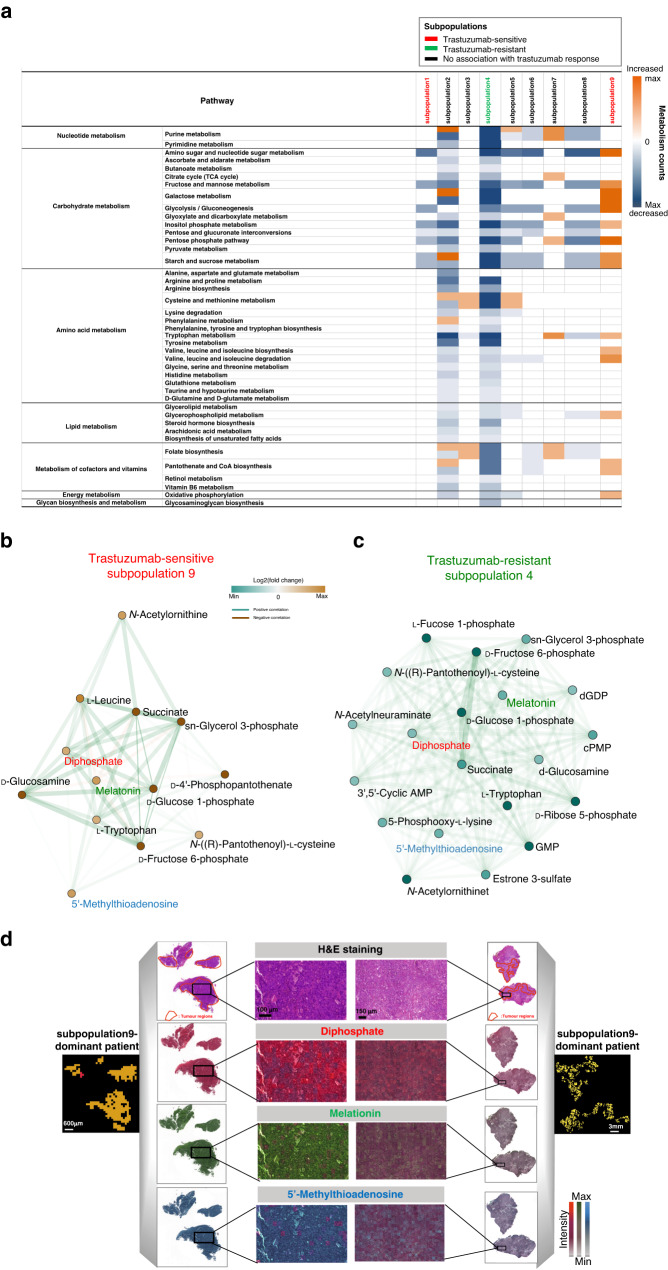
Identification of discriminative metabolites and metabolic pathways in the tumour metabolic subpopulations. **a** Pixel-wise Mann–Whitney *U*-test analysis was performed for each metabolite. Coloured squares represent counts of altered metabolites: orange ones indicate increased metabolites; blue ones indicate decreased metabolites. Correlation networks of metabolites within **b** trastuzumab-sensitive subpopulation (subpopulation 9) and **c** trastuzumab-resistant subpopulation (subpopulation 4). Correlations between metabolites were calculated and filtered (adjusted *p* < 0.05). Edges represent positive (green) and negative (orange) correlations between metabolites. Node colour in the network indicates altered metabolites: orange ones indicate increased metabolites; green ones indicate decreased metabolites. **d** Representative images of metabolites from the networks of subpopulation4 and 9 are shown for diphosphate, melatonin and 5’-Methylthioadenosine. AMP: adenosine monophosphate, GMP: guanosine monophosphate, dGDP: deoxyguanosine diphosphate, cPMP: cyclic pyranopterin monophosphate.

Next, Mann–Whitney *U*-test analysis was performed to determine which metabolites differentiated the tumour subpopulations. The metabolites responsible for these distinct tumour subpopulations were subjected to pathway analysis. Tumour metabolic heterogeneity among distinct subpopulations could be observed at the level of pathway analysis. Figure 5a summarises the discriminative pathways of each of the nine subpopulations. Classes of metabolic pathways with high number of variations are carbohydrate metabolism, nucleotide metabolism, lipid metabolism, amino acid metabolism, and metabolism of cofactors and vitamins. It is noteworthy that the trastuzumab-resistant subpopulation (subpopulation 4) exhibits profound changes in downregulated pathways; the most significant pathways are related to nucleotide metabolism, carbohydrate metabolism and amino acid metabolism. By contrast, the trastuzumab-sensitive subpopulation (subpopulation 9) shows profound changes in upregulated pathways; the most significant pathways are related to carbohydrate metabolism and amino acid metabolism.

To gain deeper insight into the molecules’ processes and events that play a role in tumour cells, and which are related to patients’ trastuzumab response, we performed a metabolic network analysis evaluating the co-localisation pattern of the metabolites in the trastuzumab-resistant subpopulation 4 and trastuzumab-sensitive subpopulaiton 9. The spatial correlation networks illustrated in Fig. 5b, c reveal the correlation of functionally interconnected metabolites in the two subpopulations. The dense cluster in the trastuzumab-resistant subpopulation 4 indicates a strong correlation of metabolites involved in the nucleotide metabolism, such as GMP, d-Ribose 5-phosphate and Precursor Z (Fig. 5b). For the trastuzumab-sensitive subpopulaiton 9, there are strong correlations of metabolites involved in the carbohydrate metabolism, such as d-Glucosamine, d-Fructose 6-phosphate and d-Glucose 1-phosphate (Fig. 5c). Representative images of diphosphate, melatonin and 5’-Methylthioadenosine from the networks of subpopulation4 and 9 are shown in Fig. 5d. Both metabolite networks are related with sn-glycerol 3-phosphate, melatonin, 5’-Methylthioadenosine and diphosphate, indicating their importance in associating with trastuzumab therapy response in HER2-positive GC.

## Discussion

In the present study, we discovered heterogeneity in a series of patients with HER2-positive advanced GC based on tissue metabolomics. We defined nine distinct metabolic subpopulations. Of the nine subpopulations, two subpopulations were associated with favourable prognosis and trastuzumab sensitivity, and one subpopulation was associated with poor prognosis and trastuzumab resistance. Additionally, tumour metabolic heterogeneity was associated with prognosis and trastuzumab response. To our knowledge, this study is the first to investigate the impact of metabolic heterogeneity on the trastuzumab treatment efficacy and survival in HER2-positive advanced GC. A higher degree of tumour metabolic heterogeneity associated with a better prognosis and trastuzumab sensitivity. This observation is in line with previous studies [12, 38]. One study described the high incidence of intratumoural HER2 heterogeneity in a large series of 322 patients with GC in detail by performing HER2 immunohistochemistry (IHC) and fluorescence in situ hybridisation (FISH) and evaluating the gene copy number individually in distinct areas with different IHC staining intensity. In addition, they further revealed that HER2 heterogeneous positivity was associated with longer survival than the homogeneous [12]. Another study consistently reported proteomic heterogeneity and their positive correlation with prognosis in HER2-positive breast cancer patients treated with trastuzumab [38]. Moreover, they revealed that high heterogeneity of tumours could reflect the presence of heterotypic components including infiltrating immune cells, which facilitated the response to treatment [38]. This could be the possible explanation of the observed correlation between a higher metabolic heterogeneity and a better outcome in HER2-positive advanced GC in the present study. Taken together, those studies together with us demonstrated the association of tumour heterogeneity of the molecular expression with trastuzumab response, indicating that molecular heterogeneity should be taken into consideration when clinical therapeutic decision of trastuzumab is made. The most significant pathways among nine tumour subpopulations were related to nucleotide metabolism and carbohydrate metabolism, which are revealed to be highly spatially organised and could be visualised as different molecularly defined regions. Major changes in nucleotides and nucleotide metabolism have been linked to patient survival. Typically, cancer cells have deactivated crucial DNA damage response signalling routes and often rewire their metabolism and energy production networks [39, 40]. Anabolic metabolism of DNA was identified as an important downstream effect of the HER2 oncogene in breast cancer [41]. In GC, one study characterised GC with metabolomic features and identified three tumour-specific subtypes. One tumour-specific subtype comprised enriched DNA metabolism, and it predicted a benefit when initiating trastuzumab therapy [20]. Another study identified DNA metabolism as a factor influencing response to HER2-targeted trastuzumab therapy, and the changes in DNA metabolism found in patient tissues were validated in a HER2-positive/sensitive and HER2-positive/resistant GC cell model [21]. The nucleotide metabolites GDP and GMP showed significant effect on survival in the GC patients treated with trastuzumab therapy [21]. This study is consistent and found that the subpopulation with downregulated nucleotide metabolism (subpopulation 4) was associated with a resistance to trastuzumab therapy.

Meanwhile, correlated metabolites within the trastuzumab-sensitive subpopulaiton 9 comprise different carbohydrate compounds, such as d-Glucosamine, d-Fructose 6-phosphate and d-Glucose 1-phosphate. These compounds are involved in different pathways contributing to tumour cell survival [42, 43]. d-glucosamine and and its derivatives have shown their anti-tumour effects on cell proliferation, cell death and angiogenesis in human bodies, although the precise function and mechanism remains to be clarified [43]. Additionally, carbohydrate metabolism is the major HER2-related altered metabolic pathway, and the association of glucose metabolism with HER2-positive breast cancer was confirmed [44, 45]. Gluconeogenesis in HER2-positive breast cancer was upregulated for energy supply, resulting in enriched consumption of related amino acids [46]. In particular, previous studies support our observation in the metabolite networks that the metabolites succinate, sn-glycerol 3-phosphate, 5’-Methylthioadenosine and diphosphate showed significant importance in distinguishing trastuzumab-sensitive and trastuzumab-resistant patients, which can be interpreted as the potential biomarkers for the trastuzumab therapy response [20, 21]. However, these new potential metabolite biomarkers and their related metabolisms have not yet fully investigated in GC. A greater understanding of these metabolite biomarkers in the future could reveal detailed insights into the molecular changes underlying GC disease, metabolic responses to treatments, and mechanisms leading to trastuzumab therapy response.

One challenge in identifying metabolic heterogeneity for their association with trastuzumab response is the limited number of tumour samples. All patients must have HER2 positivity, trastuzumab treatment, and adequate follow-up. Industry-sponsored controlled clinical trials do exist; however, the availability of these studies for independent research is unfortunately limited. Although the number of tumour samples is limited in the current study as well, the samples and associated data still offer some advantages. The tissue specimens in this study were collected from many sites. Furthermore, HER2 testing was centrally performed with the highest quality standards [34]. This ensured that the inclusion criteria were validated for each tumour sample. In conclusion, we demonstrated the importance of considering tumour metabolic heterogeneity in HER2-positive advanced GC for optimising patient management. Consequently, tumour metabolic heterogeneity showed an impact on trastuzumab efficacy and patient outcomes. These findings should be validated in larger independent cohorts, and additional molecular correlative analysis are warranted.

### Supplementary information

Supplementary Fig. 1

## Data Availability

The raw spectra data that support the findings of this study are available online (https://figshare.com/articles/dataset/MALDI-IMS_spectra_in_HER2_sup_sup_gastric_cancer_tissues/24347002).

## Code availability

Codes are provided and publicly accessible under https://github.com/mango1213/Metabolic-heterogeneity-repository.

## References

[1] Sung H, Ferlay J, Siegel RL, Laversanne M, Soerjomataram I, Jemal A (2021). Global cancer statistics 2020: GLOBOCAN estimates of incidence and mortality worldwide for 36 cancers in 185 countries. CA Cancer J Clin.

[2] Bang YJ, Van Cutsem E, Feyereislova A, Chung HC, Shen L, Sawaki A (2010). Trastuzumab in combination with chemotherapy versus chemotherapy alone for treatment of HER2-positive advanced gastric or gastro-oesophageal junction cancer (ToGA): a phase 3, open-label, randomised controlled trial. Lancet.

[3] Gomez-Martin C, Plaza JC, Pazo-Cid R, Salud A, Pons F, Fonseca P (2013). Level of HER2 gene amplification predicts response and overall survival in HER2-positive advanced gastric cancer treated with trastuzumab. J Clin Oncol.

[4] Burrell RA, McGranahan N, Bartek J, Swanton C (2013). The causes and consequences of genetic heterogeneity in cancer evolution. Nature.

[5] Renovanz M, Kim EL (2014). Intratumoral heterogeneity, its contribution to therapy resistance and methodological caveats to assessment. Front Oncol.

[6] Cioce M, Sacconi A, Pass HI, Canino C, Strano S, Blandino G (2021). Insights into intra-tumoral heterogeneity: transcriptional profiling of chemoresistant MPM cell subpopulations reveals involvement of NFkB and DNA repair pathways and contributes a prognostic signature. Int J Mol Sci.

[7] Hajjaji N, Abbouchi M, Nguyen LA, Charles S, Leclercq S, Bertin D (2020). A novel proteomic mass spectrometry-based approach to reveal functionally heterogeneous tumor clones in breast cancer metastases and identify clone-specific drug targets. J Clin Oncol.

[8] Ho SWT, Tan P (2019). Dissection of gastric cancer heterogeneity for precision oncology. Cancer Sci.

[9] Pectasides E, Stachler MD, Derks S, Liu Y, Maron S, Islam M (2018). Genomic heterogeneity as a barrier to precision medicine in gastroesophageal adenocarcinoma. Cancer Discov.

[10] Zhang M, Hu S, Min M, Ni Y, Lu Z, Sun X (2021). Dissecting transcriptional heterogeneity in primary gastric adenocarcinoma by single cell RNA sequencing. Gut.

[11] Gambardella V, Fleitas T, Tarazona N, Cejalvo JM, Gimeno-Valiente F, Martinez-Ciarpaglini C (2019). Towards precision oncology for HER2 blockade in gastroesophageal adenocarcinoma. Ann Oncol.

[12] Lee HE, Park KU, Yoo SB, Nam SK, Park DJ, Kim HH (2013). Clinical significance of intratumoral HER2 heterogeneity in gastric cancer. Eur J Cancer.

[13] Yagi S, Wakatsuki T, Yamamoto N, Chin K, Takahari D, Ogura M (2019). Clinical significance of intratumoral HER2 heterogeneity on trastuzumab efficacy using endoscopic biopsy specimens in patients with advanced HER2 positive gastric cancer. Gastric Cancer.

[14] Balluff B, Frese CK, Maier SK, Schone C, Kuster B, Schmitt M (2015). De novo discovery of phenotypic intratumour heterogeneity using imaging mass spectrometry. J Pathol.

[15] Chao J, Bedell V, Lee J, Li MS, Chu P, Yuan YC (2020). Association between spatial heterogeneity within nonmetastatic gastroesophageal adenocarcinomas and survival. JAMA Netw Open.

[16] Abdelmoula WM, Balluff B, Englert S, Dijkstra J, Reinders MJ, Walch A (2016). Data-driven identification of prognostic tumor subpopulations using spatially mapped t-SNE of mass spectrometry imaging data. Proc Natl Acad Sci USA.

[17] Zhang Z, Bao C, Jiang L, Wang S, Wang K, Lu C (2022). When cancer drug resistance meets metabolomics (bulk, single-cell and/or spatial): Progress, potential, and perspective. Front Oncol.

[18] Wei S, Liu L, Zhang J, Bowers J, Gowda GA, Seeger H (2013). Metabolomics approach for predicting response to neoadjuvant chemotherapy for breast cancer. Mol Oncol.

[19] Yang K, Zhang F, Han P, Wang ZZ, Deng K, Zhang YY (2018). Metabolomics approach for predicting response to neoadjuvant chemotherapy for colorectal cancer. Metabolomics.

[20] Wang J, Kunzke T, Prade VM, Shen J, Buck A, Feuchtinger A (2022). Spatial metabolomics identifies distinct tumor-specific subtypes in gastric cancer patients. Clin Cancer Res.

[21] Kunzke T, Holzl FT, Prade VM, Buck A, Huber K, Feuchtinger A (2021). Metabolomic therapy response prediction in pretherapeutic tissue biopsies for trastuzumab in patients with HER2-positive advanced gastric cancer. Clin Transl Med.

[22] Prade VM, Kunzke T, Feuchtinger A, Rohm M, Luber B, Lordick F (2020). De novo discovery of metabolic heterogeneity with immunophenotype-guided imaging mass spectrometry. Mol Metab.

[23] Balluff B, Rauser S, Ebert MP, Siveke JT, Hofler H, Walch A (2012). Direct molecular tissue analysis by MALDI imaging mass spectrometry in the field of gastrointestinal disease. Gastroenterology.

[24] Aichler M, Walch A (2015). MALDI imaging mass spectrometry: current frontiers and perspectives in pathology research and practice. Lab Invest.

[25] Shen J, Sun N, Wang J, Zens P, Kunzke T, Buck A (2023). Patterns of carbon-bound exogenous compounds impact disease pathophysiology in lung cancer subtypes in different ways. ACS Nano.

[26] Addie RD, Balluff B, Bovee JV, Morreau H, McDonnell LA (2015). Current state and future challenges of mass spectrometry imaging for clinical research. Anal Chem.

[27] Oppenheimer SR, Mi D, Sanders ME, Caprioli RM (2010). Molecular analysis of tumor margins by MALDI mass spectrometry in renal carcinoma. J Proteome Res.

[28] Kulbe H, Klein O, Wu Z, Taube ET, Kassuhn W, Horst D (2020). Discovery of prognostic markers for early-stage high-grade serous ovarian cancer by MALDI-imaging. Cancers.

[29] Cornett DS, Reyzer ML, Chaurand P, Caprioli RM (2007). MALDI imaging mass spectrometry: molecular snapshots of biochemical systems. Nat Methods.

[30] Shen J, Sun N, Zens P, Kunzke T, Buck A, Prade VM (2022). Spatial metabolomics for evaluating response to neoadjuvant therapy in non-small cell lung cancer patients. Cancer Commun.

[31] Deininger SO, Ebert MP, Futterer A, Gerhard M, Rocken C (2008). MALDI imaging combined with hierarchical clustering as a new tool for the interpretation of complex human cancers. J Proteome Res.

[32] Jones EA, Schmitz N, Waaijer CJ, Frese CK, van Remoortere A, van Zeijl RJ (2013). Imaging mass spectrometry-based molecular histology differentiates microscopically identical and heterogeneous tumors. J Proteome Res.

[33] Wang J, Sun N, Kunzke T, Shen J, Zens P, Prade VM (2023). Spatial metabolomics identifies distinct tumor-specific and stroma-specific subtypes in patients with lung squamous cell carcinoma. NPJ Precis Oncol.

[34] Haffner I, Schierle K, Raimundez E, Geier B, Maier D, Hasenauer J (2021). HER2 expression, test deviations, and their impact on survival in metastatic gastric cancer: results from the prospective multicenter VARIANZ study. J Clin Oncol.

[35] Ly A, Buck A, Balluff B, Sun N, Gorzolka K, Feuchtinger A (2016). High-mass-resolution MALDI mass spectrometry imaging of metabolites from formalin-fixed paraffin-embedded tissue. Nat Protoc.

[36] Simpson EH (1949). Measurement of diversity. Nature.

[37] Shannon P, Markiel A, Ozier O, Baliga NS, Wang JT, Ramage D (2003). Cytoscape: a software environment for integrated models of biomolecular interaction networks. Genome Res.

[38] Gawin M, Kurczyk A, Niemiec J, Stanek-Widera A, Grela-Wojewoda A, Adamczyk A (2021). Intra-tumor heterogeneity revealed by mass spectrometry imaging is associated with the prognosis of breast cancer. Cancers.

[39] Lord CJ, Ashworth A (2012). The DNA damage response and cancer therapy. Nature.

[40] Dang CV (2012). Links between metabolism and cancer. Genes Dev.

[41] Nikolai BC, Lanz RB, York B, Dasgupta S, Mitsiades N, Creighton CJ (2016). HER2 signaling drives DNA anabolism and proliferation through SRC-3 phosphorylation and E2F1-regulated genes. Cancer Res.

[42] Liu H, Heaney AP (2011). Refined fructose and cancer. Expert Opin Ther Targets.

[43] Zahedipour F, Dalirfardouei R, Karimi G, Jamialahmadi K (2017). Molecular mechanisms of anticancer effects of glucosamine. Biomed Pharmacother.

[44] Zhang D, Tai LK, Wong LL, Chiu L-L, Sethi SK, Koay ES (2005). Proteomic study reveals that proteins involved in metabolic and detoxification pathways are highly expressed in HER-2/neu-positive breast cancer. Mol Cell Proteom.

[45] Walsh AJ, Cook RS, Manning HC, Hicks DJ, Lafontant A, Arteaga CL (2013). Optical metabolic imaging identifies glycolytic levels, subtypes, and early-treatment response in breast cancer. Cancer Res.

[46] Fong MY, Zhou W, Liu L, Alontaga AY, Chandra M, Ashby J (2015). Breast-cancer-secreted miR-122 reprograms glucose metabolism in premetastatic niche to promote metastasis. Nat Cell Biol.

