# Supplementary material for: Metabolic heterogeneity affects trastuzumab response and survival in HER2-positive advanced gastric cancer
Source: Br J Cancer. 2024 Jan 24;130(6):1036–45. doi: 10.1038/s41416-023-02559-6 (PMC10951255; doi:10.1038/s41416-023-02559-6)
Supplement: Supplementary file 1 — Supplementary Fig. 1 [file 41416_2023_2559_MOESM1_ESM.docx]

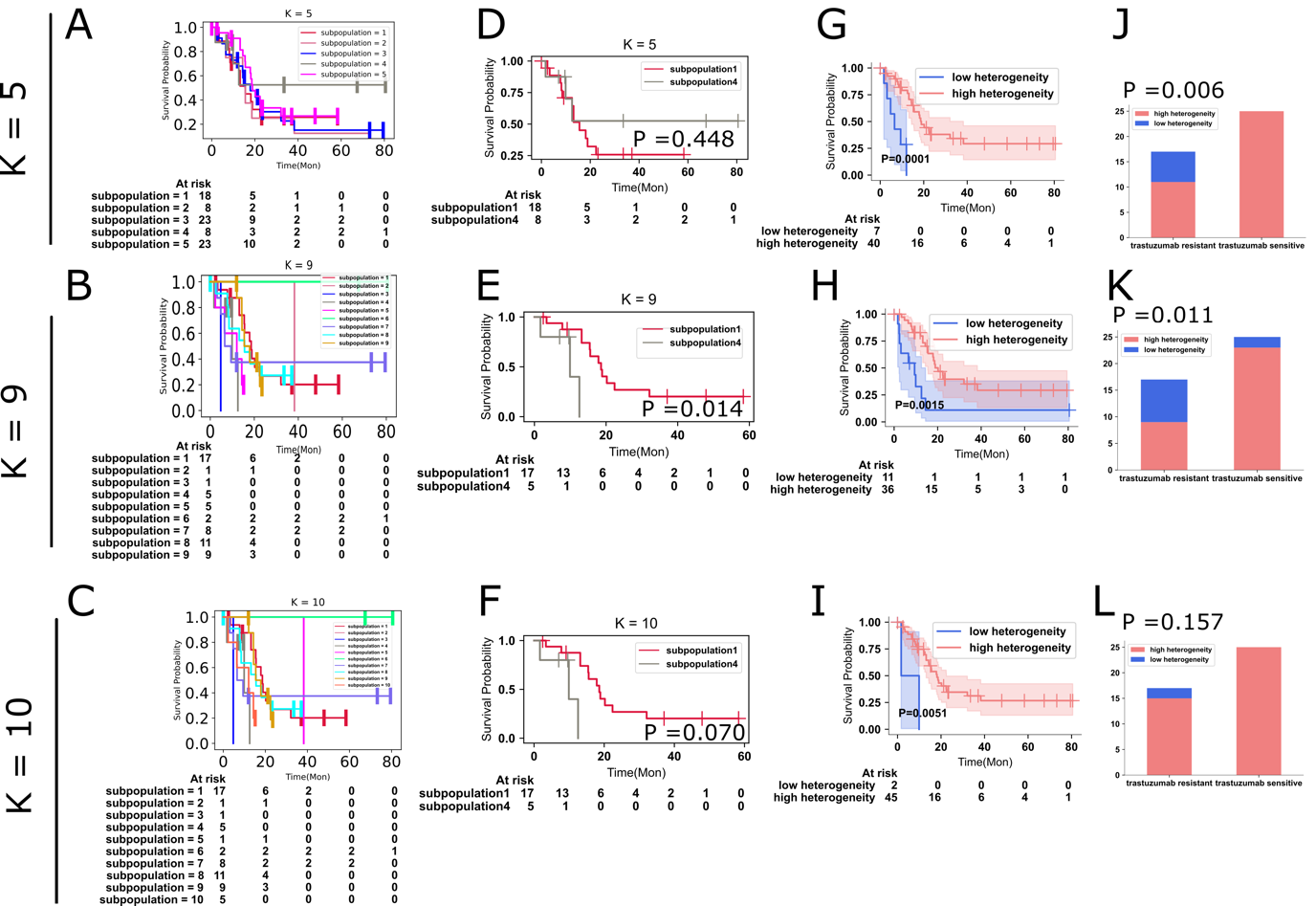


**Supplementary Fig. 1 Kaplan-Meier survival curves found for three values of K for the gastric cancer MSI dataset: K = 5, top row; K = 9, middle row; K = 10, bottom row.** Survival analysis of all tumor metabolic subpopulations in Kaplan–Meier curves of (A) K=5, (B) K=9 and (C) K=10. Survival differences between subpopulations 1 and 4 of (D) K=5, (E) K=9 and (F) K=10. Survival difference between patients with high metabolic heterogeneity levels and patients with low metabolic heterogeneity levels of (G) K=5, (H) K=9 and (I) K=10. Numbers of high heterogeneity and low heterogeneity patients in trastuzumab-sensitive and trastuzumab-resistant patients of (J) K=5, (K) K=9 and (L) K=10.
